# Dietary grape seed proanthocyanidins (GSPs) improve weaned intestinal microbiota and mucosal barrier using a piglet model

**DOI:** 10.18632/oncotarget.13450

**Published:** 2016-11-18

**Authors:** Meng Han, Peixia Song, Chang Huang, Arash Rezaei, Shabnam Farrar, Michael A. Brown, Xi Ma

**Affiliations:** ^1^ State Key Laboratory of Animal Nutrition, China Agricultural University, Beijing, China; ^2^ School of Medicine, University of Central Florida, Orlando, FL, USA; ^3^ College of Dental Medicine, Midwestern University, Downers Grove IL, USA; ^4^ Department of Animal Science, Oklahoma State University, Stillwater, OK, USA; ^5^ Department of Internal Medicine, Department of Biochemistry, Center for Autophagy Research, University of Texas Southwestern Medical Center, Dallas, TX, USA

**Keywords:** grape seed proanthocyanidins, antibiotic alternative, bacterial composition and distribution, intestinal mucosal barrier, oxidative stress, Immunology and Microbiology Section, Immune response, Immunity

## Abstract

Proanthocyanidins have been suggested as an effective antibiotic alternative, however their mechanisms are still unknown. The present study investigated the effects of grape seed proanthocyanidins on gut microbiota and mucosal barrier using a weaned piglet model in comparison with colistin. Piglets weaned at 28 day were randomly assigned to four groups treated with a control ration, or supplemented with 250 mg/kg proanthocyanidins, kitasamycin/colistin, or 250 mg/kg proanthocyanidins and half-dose antibiotics, respectively. On day 28, the gut chyme and tissue samples were collected to test intestinal microbiota and barrier function, respectively. Proanthocyanidins treated piglets had better growth performance and reduced diarrhea incidence (*P* < 0.05), accompanied with decreased intestinal permeability and improved mucosal morphology. Gene sequencing analysis of 16S rRNA revealed that dietary proanthocyanidins improved the microbial diversity in ileal and colonic digesta, and the most abundant OTUs belong to *Firmicutes* and *Bacteroidetes spp*. Proanthocyanidins treatment decreased the abundance of *Lactobacillaceae*, and increased the abundance of *Clostridiaceae* in both ileal and colonic lumen, which suggests that proanthocyanidins treatment changed the bacterial composition and distribution. Administration of proanthocyanidins increased the concentration of propionic acid and butyric acid in the ileum and colon, which may activate the expression of GPR41. In addition, dietary proanthocyanidins improved the antioxidant indices in serum and intestinal mucosa, accompanied with increasing expression of barrier occludin. Our findings indicated that proanthocyanidins with half-dose colistin was equivalent to the antibiotic treatment and assisted weaned animals in resisting intestinal oxidative stress by increasing diversity and improving balance of gut microbes.

## BACKGROUND

Weaning is often stressful for infants and young animals and accompanied by intestinal, microbial, and immunological changes in the digestive tract, which cause diarrhea, growth performance reduction, and other diseases, including mortality [1-2]. Antibiotics have been widely used to control weaning diarrhea and have been adequately effective. However, the overuse of antibiotics has been associated with increasing adverse side effects especially antibiotic resistance [3-4]. Colistin is increasingly used as an antibiotic of last resort for the treatment of carbapenem-resistant Gram-negative infections. However, resistance to colistin, encoded by the plasmid-borne gene mcr-1, was first identified in animal and clinical samples from China in November 2015 and has subsequently been reported from numerous other countries [5-6]. Therefore, identification of alternatives to antibiotics is imperative.

The vast majority of antibiotic substitutes produce inconsistent results and rarely have the same effectiveness as antibiotics [7]. Recent evidence indicated the beneficial effects of grape seed proanthocyanidins (GSPs) on the mammalian animals' gut as an effective antibiotic alternative [8]. GSPs are primarily known for their antioxidant activity. The strong antioxidant effect of GSPs is due to their effective hydrogen donation, as well as effective delocalization of an unpaired electron. Dietary flavonoids from plant foods have gained considerable attention as a dietary additive to improve the health status and growth [9]. However, the detailed mechanism of how the GSPs improve the gut functions is still unknown.

The mammalian digestive tract is colonized by a dense, dynamic and highly complex community of microorganisms composed mainly of bacteria, whose total number exceeds 10^14^ cells with thousands of individual strains [10]. The intestine, which harbors a complex microbiota and a highly evolved mucosal immune system, plays an essential role in host health [11]. A balanced gut microflora plays a vital role in metabolism, pathogen resistance, and immune development [12-13]. The high concentration of short-chain carbohydrates and proteins in the small intestine promotes the growth of bacteria (e.g. *Proteobacteria* and *Lactobacillales)* which can metabolize these carbohydrate and protein molecules [14]. In the large intestine, most of the nutrients available for bacteria are derived from indigestible or resistant carbohydrates as well as undigested protein in the diet. These dietary nutrients then undergo microbial fermentation resulting in the production of metabolites, such as volatile fatty acids (VFAs) and biogenic amines [15-16]. Both VFAs and polyamimes can improve the expression of intestinal tight junction proteins, which are the important constitutes of the intestinal barrier, and help resist the invasion of pathogens [17-19].

In our previous study, the beneficial effects of GSPs as alternatives to antibiotics have been confirmed in the control of weaning diarrhea using rat models [20]. In the present study, using 16S RNA sequencing microbiome analysis, our objective was to identify the potential mechanisms by which dietary GSPs improve gut function and reduce weaning diarrhea *via* modulating intestinal microbiota and barrier.

## RESULTS

### Dietary GSPs improved growth performance and decreased diarrhea incidence

Dietary supplementation with GSPs, antiobiotics, and GSPs + antibiotics improved the average daily weight gain (ADG) of weaned piglets (*P* < 0.05), and the ratio of average daily feed intake (ADFI) to ADG (F/G ratio) when compared with the negative control (NC) group (Table 1). These data indicated that dietary GSPs can improve the growth performance, and dietary supplementation with GSPs can at least effectively replace half of the dosage of antibiotics. There were no important differences in ADFI among groups, which suggests that GSPs may not affect appetite.

During the first, second, and fourth week, the GSPs group decreased the incidence of piglet diarrhea compared with the NC group (*P* < 0.05), similar to the PC group (Table 1). The AGS group also decreased diarrhea incidence (*P* < 0.05). These data suggested that the improved growth performance by GSPs may be associated with decreased diarrhea incidence.

**Table 1:** Effects of dietary grape seed proanthocyanidins on growth performance and diarrhea incidence^1^,^2^,^3^

Variable	NC	GSPs	AGS	PC
Growth performance				
Average weight gain, g/d	467 ± 15^b^	494 ± 18^a^	487 ± 16^a^	499 ± 19^a^
Average feed intake, g/d	862 ± 45^a^	835 ± 57^ab^	839 ± 24^b^	893 ± 21^a^
Feed: Gain, g/g	1.84 ± 0.05^a^	1.69 ± 0.05^b^	1.72 ± 0.06^b^	1.73 ± 0.04^b^
Weekly diarrhea incidence,^2^ %				
First week	37.1^a^	23.7^b^	22.3^b^	21.9^b^
Second week	24.3^a^	16.7^b^	16.4^b^	16.8^b^
Fourth week	34.9^a^	23.9^b^	28.3^ab^	23.1^b^

1 Values are means ± SEMs, n = 30 for average weight gain, average feed intake and Feed: Gain. Different superscript within a row means significantly different (*P* < 0.05).

2 Weekly diarrhea incidence (%) = the number of pigs with diarrhea in each pen × diarrhea days ÷ (total number of piglets × 7 d) × 100.

3 NC, negative control; GSPs, grape seed proanthocyanidins; AGS, reduced antibiotics + GSPs; PC, positive control.

### Dietary GSPs decreased the intestinal permeability and improved the intestinal mucosal morphology

**Figure 1:**
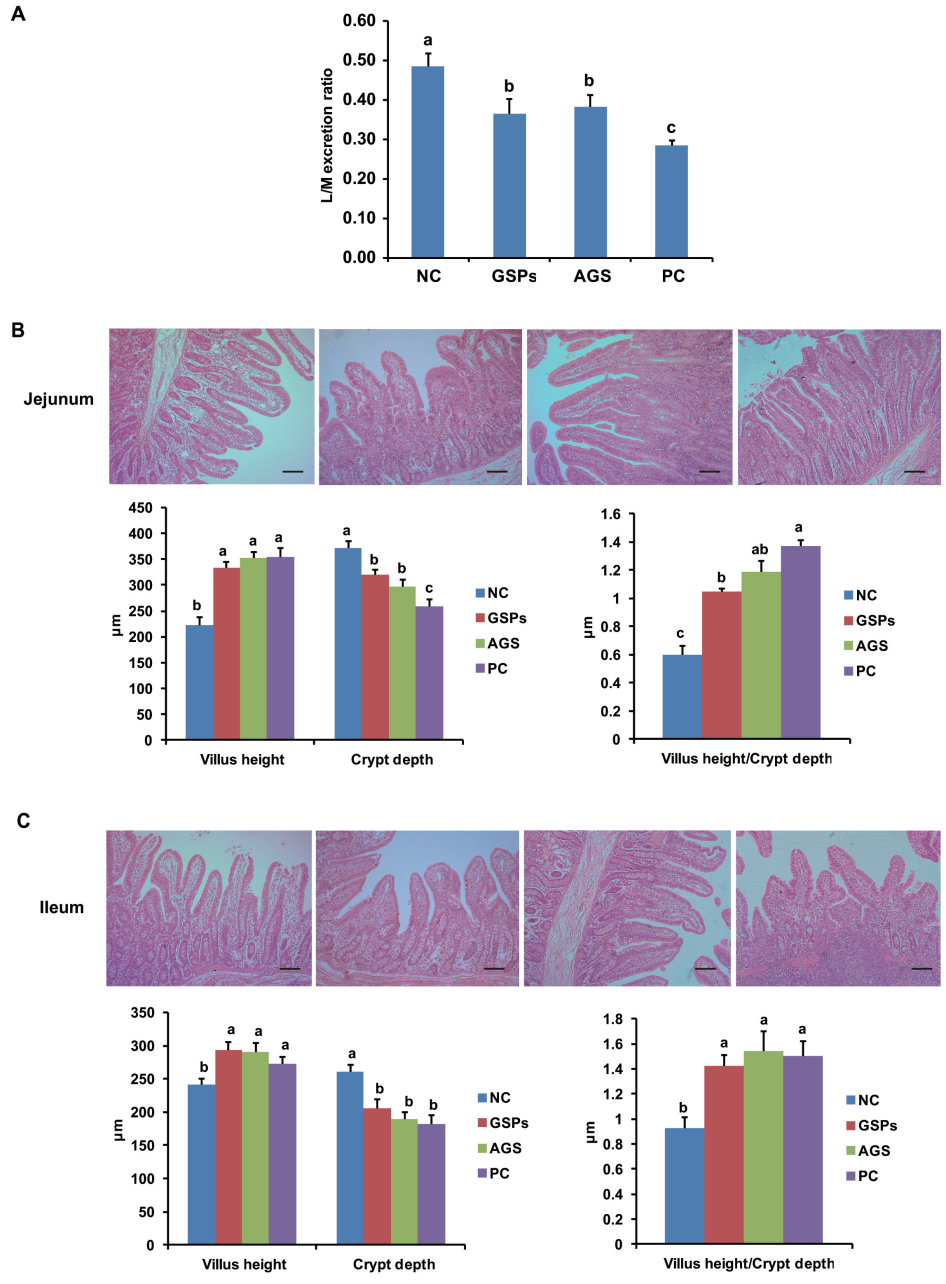
Effects of dietary grape seed proanthocyanidins on intestinal permeability and intestinal morphology **A.** Intestinal permeability measured by lactulose/mannitol excretion ratio (L/M ratio) in each group. Values are means ± SEMs, *n* = 6. Different superscript within each group means significantly different (*P* < 0.05). **B.**-**C.** The mucosa in the segments of **B.** jejunum and **C.** ileum of the selected piglets in each group were isolated, fixed in 4% paraformaldehyde for 24 h, and then embedded in paraffin wax. Sections of 4 μm were cut and stained with hematoxylin and eosin. Representative photos were taken and villus height and crypt depth were measured. Values are means ± SEMs, *n* = 6. Different superscript within each group means significantly different (*P* < 0.05). NC, negative control; GSPs, grape seed proanthocyanidins; AGS, reduced antibiotics + GSPs; PC, positive control.

To identify the reason for improved growth performance and reduced diarrhea occurrence, the intestinal permeability was monitored using the lactulose/mannitol test and the intestinal mucosal morphology was determined. The intestinal permeability in the GSPs, AGS and PC groups significantly decreased when compared with that of the NC group (*P* < 0.05) (Figure 1A). This is consistent with the results of growth performance. In addition, the GSPs, AGS and PC treatments increased villus height of the jejunum and ileum (*P* < 0.05), while crypt depth in the three treatments significantly decreased compared with the control group (*P* < 0.05). Therefore, the ratio of villus height to crypt depth ratio in jejunum and ileum significantly increased by GSPs, AGS and PC treatments (*P* < 0.05) (Figure 1B-1C). These data indicated that dietary GSPs can improve the intestinal permeability and the mucosal morphology, which may explain a decrease in diarrhea incidence.

### Dietary GSPs increased the intestinal bacterial abundance and diversity in digesta

To further study the mechanism of dietary GSPs on improved intestinal mucosal morphology and permeability, the intestinal bacterial abundance and diversity in digesta were determined. In total, 453267 reads were obtained for the bacterial 16S rRNA genes by pyrosequencing. After screening these gene sequences with strict criteria (described in methods), the Chao factor analysis showed that in colon digesta GSPs group supported more abundance and diversity than the NC group and PC group, as well as the AGS group (Table 2). Similar trends were observed in ileum digesta. Additionally, the GSPs group had more abundance and diversity than the NC group and PC group but less than the AGS group. On contrast, in ileum digesta, the Shannon index was greater in the GSPs and AGS groups than the NC and PC groups. In colon digesta, the GSPs and PC treatments had positive effect on microbial abundance and diversity compared to the NC. Taken together, these results suggested that GSPs treatment can effectively improve the intestinal bacterial abundance and diversity, both in the ileal and colonic digesta, and may be a suitable substitution for dietary antibiotics.

**Table 2:** Effects of dietary grape seed proanthocyanidins on bacterial communities diversity in the ileal and colonic digesta^1^,^2^

Sample ID	Valid Sequence	Similarity score ≥ 0.97		
		OTU	Chao	Shannon
Ileum_NC	23260	41	46 (42,64)	1.81 (1.79,1.83)
Ileum_GSPs	37828	36	40 (37,55)	0.43 (0.41,0.45)
Ileum_AGS	44193	36	38 (36,48)	2.16 (2.15,2.18)
Ileum_PC	25325	38	53 (42,102)	1.36 (1.35,1.38)
Colon_NC	16310	149	168 (157,197)	2.68 (2.65,2.71)
Colon_ GSPs	41229	286	297 (290,315)	3.79 (3.76,3.81)
Colon_AGS	43560	267	275 (270,290)	3.77 (3.75,3.8)
Colon_PC	25962	280	294 (285,316)	4.17 (4.14,4.19)

1 Sequences with similarity scores greater than or equal to 0.97 were clustered into an OTU.

2 NC, negative control; GSPs, grape seed proanthocyanidins; AGS, reduced antibiotics + GSPs; PC, positive control.

### Dietary GSPs effectively improved ileal and colonic bacterial composition and distribution

The ileal and colonic bacterial community were further analyzed to identify the role of dietary GSPs on intestinal microbiota. All sequences were classified from phylum to genus, 9 different phyla and 84 different genera were identified from these samples.

**Figure 2:**
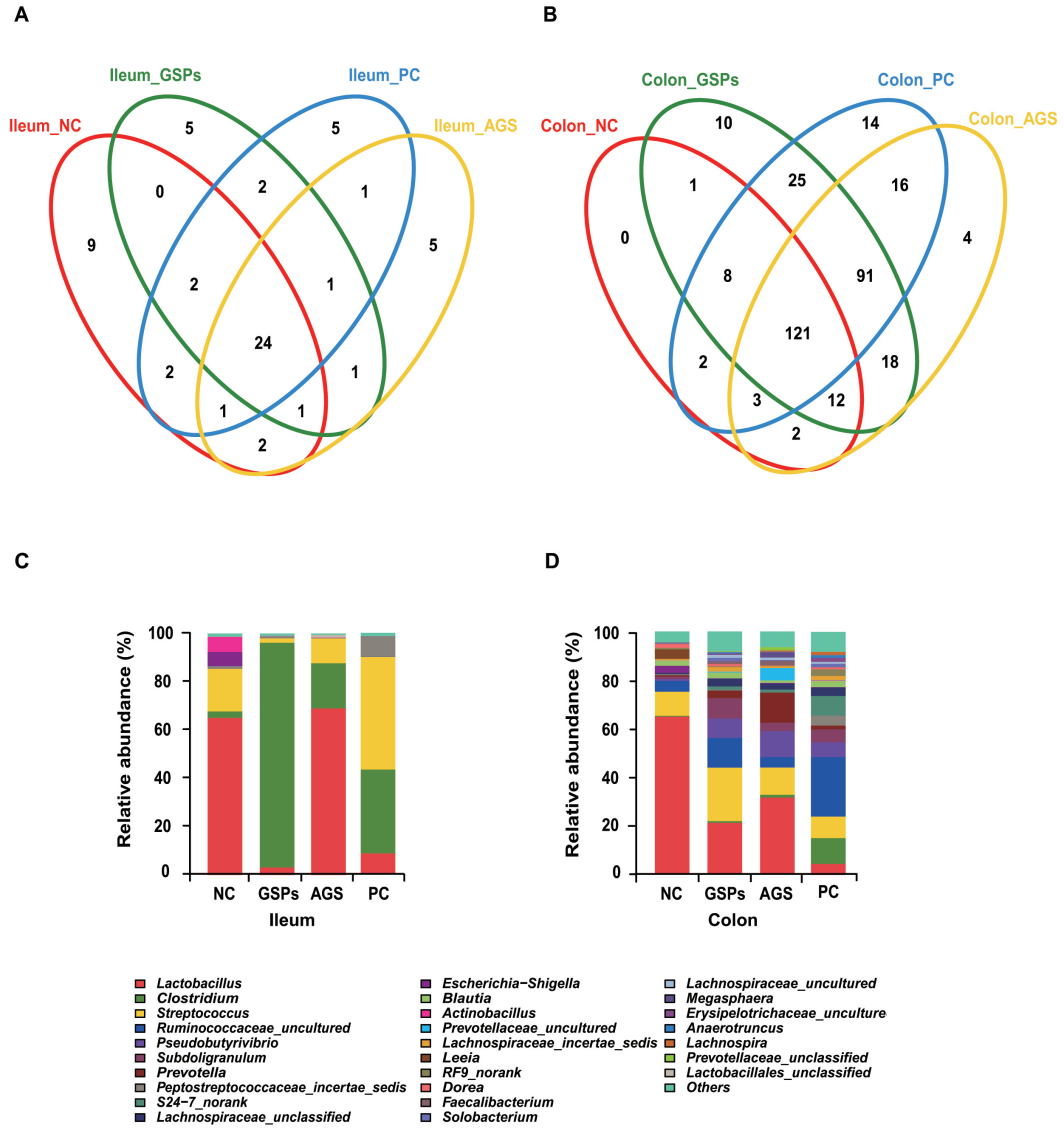
Effects of dietary grape seed proanthocyanidins on ileal and colonic bacterial community of weaned piglets **A.**-**B.** Venne diagram showing the unique and shared OTUs (3% distance level) in the different libraries of intestinal bacteria in the ileal **A.** and colonic **B.** digesta in different treatments. Relative read abundance of different bacterial families within the different communities. **C.**-**D.** Family-level distribution of luminal bacteria in the ileal **C.** and colonic **D.** digesta in different treatments. NC, negative control; GSPs, grape seed proanthocyanidins; AGS, reduced antibiotics + GSPs; PC, positive control.

16S rRNA profiles for each experimental group in the ileum and colon were very dissimilar even in phylum level distributions. The most abundant OTUs associated with the colon digesta from the GSPs group were *Firmicutes* (78.25%) and *Bacteroidetes* (20.34%), compared with the NC group (*P* < 0.05). The OTU composition and abundance were relatively similar between the colon and ileum content in the same treatment groups, such as the two AGS groups, and the NC and PC group (data not shown). The *Firmicutes* and *Bacteroidetes* bacteria in the colon and ileum digesta were further investigated to identify classes, genera and species. Each experimental group in the ileum and colon had 24 and 121 OTUs in common, respectively (Figure 2A-2B). Within these two dominating phyla, *Bacilli*, *Clostridia* and *Bacteroidia* represented the most abundant classes common to the eight libraries. For *Actinobacteria*, *Chlamydiae*, *Chloroplast*, *Negativicutes* and *Coriobacteriia*, OTUs common to the eight libraries were in low abundance.

In the analysis of the microbiota composition down to family levels for all treatments, 36 taxa were identified. In the NC treatment in the ileal lumen, the majority of classifiable sequences belonging to *Lactobacillaceae* (65.1%), *Streptococcaceae* (17.6%), *Actinobacillus* (6.2%) and *Enterobacteriaceae* (5.9%) (Figure 2C), while the colonic lumen bacteria in the NC treatment were dominated by *Lactobacillaceae* (65.0%), *Streptococcaceae* (9.9%), *Ruminococcaceae* (4.5%), *Leeia* (3.9%) and *Escherichia-Shigella* (2.9%) (Figure 2D). The decrease in *Lactobacillaceae* abundance in the GSPs and PC treatments was striking: from 65.1% in the ileal lumen of untreated pigs to 3.1% with GSPs treatment and 8.7% with PC treatment (*P* < 0.05), as well as from 65.0% to 21.3%, 4.5% and 31.7% in the colonic lumen of pigs in the corresponding treatments (*P* < 0.05). Non-abundant *Clostridiaceae* became dominant in ileal lumen with dramatic increases in the abundance from 2.7% in the NC treatment to 93.0% in the GSPs treatment (*P* < 0.05), and 34.7% in the PC treatment and 18.7% in the AGS treatment. In addition, an increase in *Clostridiaceae* abundance was observed in the colonic lumen with GSPs (0.7%), PC (10.6%) and AGS (1.1%) treatments compared with the NC treatment (0.3%). In addition, certain families decreased with GSPs, PC and AGS treatments, such as *Actinobacillus*, *Streptococcus* and *Pseudobutyrivibrio* in the ileal lumen, whereas *Ruminococcaceae* and *Lachnospiraceae* increased in the colonic lumen with all treatments.

**Figure 3:**
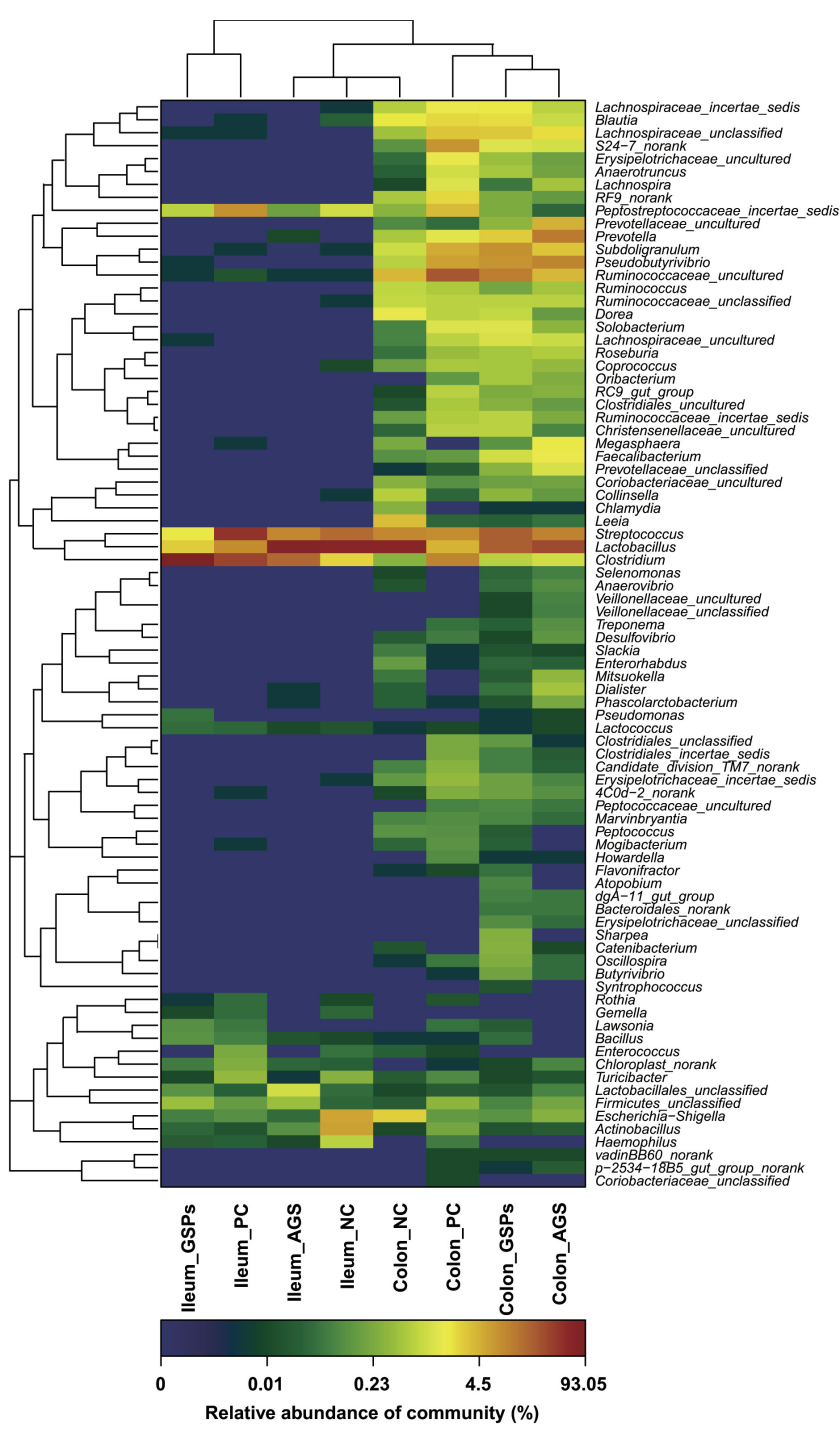
Double hierarchical dendrogram to illustrate effects of dietary grape seed proanthocyanidins on ileal and colonic bacterial community of weaned piglets The bacterial distribution among the samples bacterial phylogenetic tree was calculated using the neighbor-joining method and the relationship among samples was determined by Bray distance and the complete clustering method. The heatmap plot depicts the relative percentage of each bacteria are depicted by color intensity with the legend indicated at the bottom of the figure. Clusters based on the distance of the eight samples along the X-axis and the bacterial families along the Y-axis are indicated in the upper and left of the figure, respectively. NC, negative control; GSPs, grape seed proanthocyanidins; AGS, reduced antibiotics + GSPs; PC, positive control.

Sequence clustering within the genus level revealed that GSPs groups significantly changed the bacterial composition and distribution compared with the NC groups and PC groups (*P* < 0.05) (Figure 3). This provides solid evidence that GSPs can effectively improve bacterial community profiles. Treatment with AGS in the weaned piglets was less effective than GSPs (*P* < 0.05), which may be due to the antibiotic resistance.

### Dietary GSPs increased the concentration of propionic acid and butyric acid in intestinal digesta

To evaluate the effect of dietary GSPs on the metabolites of intestinal microbiota, VFAs in digesta were estimated. Concentrations of major VFAs were in the order of colon > ileum, as well as acetic acid > propionic acid > butyric acid > valeric acid > isobutyric acid and isovaleric acid (Table 3). The concentrations of propionic acid, butyric acid and valeric acid increased in both ileum and colon digesta in response to treatment with GSPs (*P* < 0.05) (Table 3). The VFA concentrations in the PC group decreased when compared with those in the NC group (*P* < 0.05) (Table 3). These data indicated that dietary GSPs mainly increased the concentration of propionic acid and butyric acid in both ileum and colon digesta, which suggested that propionic acid and butyric acid may mediate physiological functions of the intestinal microbiota treated with dietary GSPs.

**Table 3:** Effects of dietary grape seed proanthocyanidins on concentration of volatile fatty acids in the ileal (A) and colonic (B) digesta of piglets' samples^1^,^2^

Variable	NC	GSPs	AGS	PC
Ileum				
Acetic acid, mg/g	1.09 ± 0.11^a^	1.06 ± 0.09^a^	0.99 ± 0.08^a^	0.68 ± 0.12^b^
Propionic acid, mg/g	0.125 ± 0.005^b^	0.171 ± 0.004^a^	0.136 ± 0.007^b^	0.102 ± 0.006^c^
Butyric acid, mg/g	0.124 ± 0.007^b^	0.183 ± 0.009^a^	0.134 ± 0.006^b^	0.130 ± 0.006^b^
Valeric acid, mg/g	0.048 ± 0.009	0.055 ± 0.010	0.050 ± 0.008	0.049 ± 0.004
Isobutyric acid, mg/g	0.018 ± 0.006	0.023 ± 0.004	0.020 ± 0.007	0.020 ± 0.008
Isovaleric acid, mg/g	0.066 ± 0.008	0.070 ± 0.004	0.064 ± 0.009	0.068 ± 0.003
Colon				
Acetic acid, mg/g	3.24 ± 0.20	3.08 ± 0.15	3.11 ± 0.14	2.99 ± 0.14
Propionic acid, mg/g	1.20 ± 0.10^c^	1.91 ± 0.09^a^	1.54 ± 0.06^b^	0.97 ± 0.07^d^
Butyric acid, mg/g	0.568 ± 0.09^b^	0.746 ± 0.06^a^	0.665 ± 0.07^ab^	0.489 ± 0.05^b^
Valeric acid, mg/g	0.218 ± 0.006^a^	0.209 ± 0.006^a^	0.224 ± 0.005^a^	0.126 ± 0.008^b^
Isobutyric acid, mg/g	0.120 ± 0.007	0.132 ± 0.004	0.117 ± 0.008	0.114 ± 0.005
Isovaleric acid, mg/g	0.103 ± 0.003^a^	0.119 ± 0.007^a^	0.114 ± 0.006^a^	0.065 ± 0.005^b^

1 Values are means ± SEMs, n = 6. Different superscript within a row means significantly different (*P* < 0.05).

2 NC, negative control; GSPs, grape seed proanthocyanidins; AGS, reduced antibiotics + GSPs; PC, positive control.

### Dietary GSPs increased the abundance of GPR41 and occludin in ileum and colon

**Figure 4:**
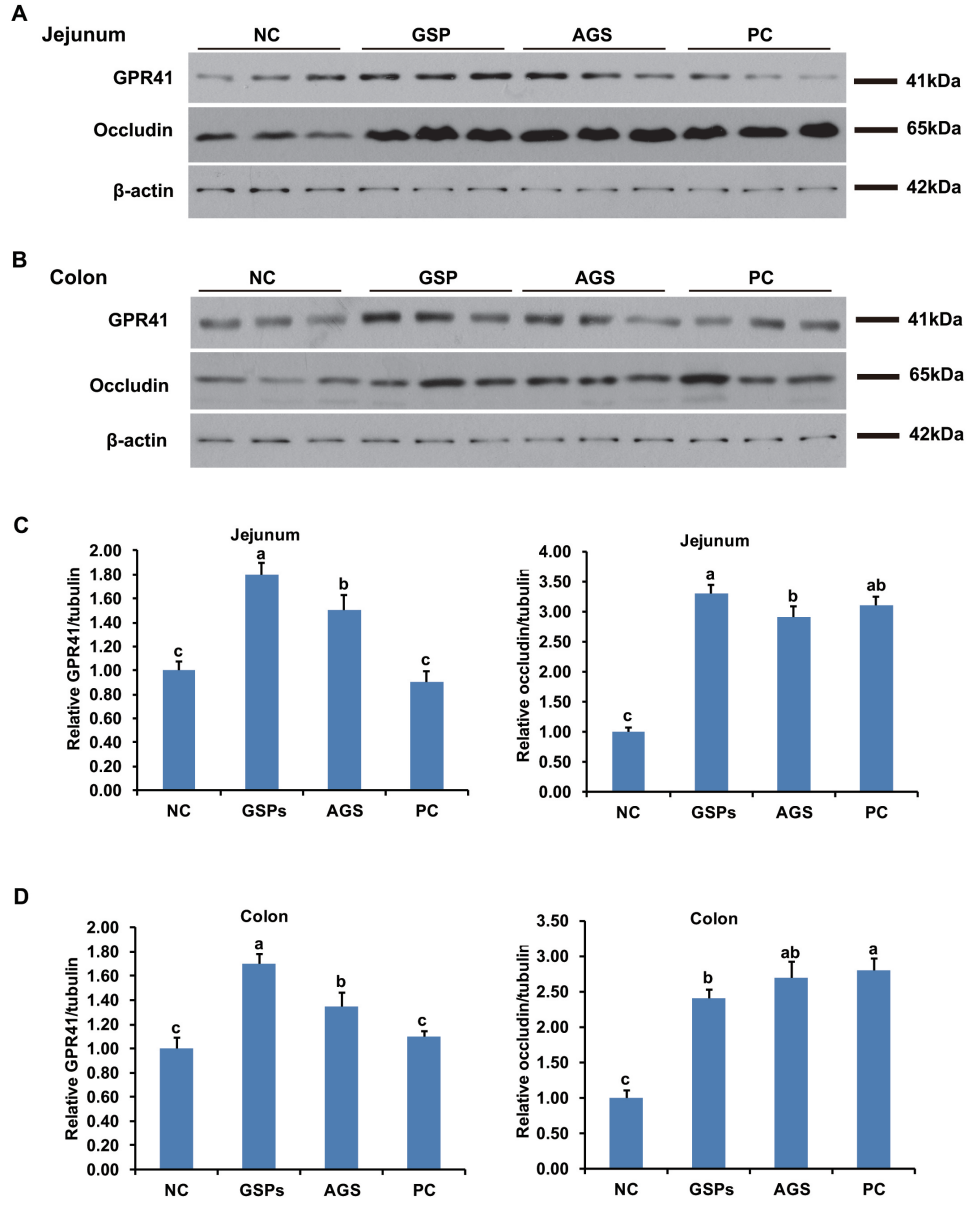
Effects of dietary grape seed proanthocyanidins on expression of mucosa G-protein coupled receptor 41 (GPR41) and barrier protein occludin **A.**-**B.** The abundance of GPR41 and occludin proteins in mucosal tissues of jejunum **A.** and colon **B.** were determined by western blot. Representative photos from three independent experiments were shown. **C.**-**D.** Quantitative analysis of **A.**-**B.** Values are means ± SEMs, *n* = 3. Different superscript within each group means significantly different (*P* < 0.05). NC, negative control; GSPs, grape seed proanthocyanidins; AGS, reduced antibiotics + GSPs; PC, positive control.

To evaluate the effect of dietary GSPs on the signal pathway of gut tissues, the expression of G-protein coupled receptor (GPR) 41, GPR43 and GPR109A, as well as mucosal barrier protein occludin, claudin-1 and zonula occludens protein-1 (ZO-1), were tested by Western blot. The abundance of GPR41 and occludin in both ileal and colonic mucosa significantly increased in the GSPs and AGS treatment groups (*P* < 0.05), when compared with the NC group (Figure 4). The abundance of GPR43 and GPR109A, as well as claudin-1 and ZO-1, had no difference in different groups in both ileal and colonic mucosa (*P* > 0.05, data not shown). These data suggested that dietary GSPs can increase the mucosal barrier function *via* activating the GPR41 expression. The activated GPR41 results were consistent with the increased propionic acid and butyric acid as described above. However, the PC treatment group only increased the expression of occludin (*P* < 0.05) (Figure 4), which suggested that the mechanism by which GSPs control diarrhea may be different from that of antibiotics.

### Dietary GSPs improved antioxidant indices in serum and intestinal mucosa

In the dietary GSPs and AGS groups, the activities of superoxide dismutase (SOD) and glutathione peroxidase (GSH-Px), as well as the content of Glutathione (GSH), increased in both the serum and intestinal mucosal tissue samples (*P* < 0.05), whereas the content of malondialdehyde (MDA) decreased (*P* < 0.05), compared with the NC group (Table 4). No statistical difference in these antioxidant indices was found between the antibiotics treatment (PC group) and the NC group (Table 4). These data suggested dietary GSPs may improve intestinal functions *via* enhanced antioxidant capacity, which differs from the apparent mechanisms of antibiotics.

**Table 4:** Effects of dietary grape seed proanthocyanidins on antioxidant indices in serum and intestinal mucosa^1^,^2^

Variable	NC	GSPs	AGS	PC
Serum				
Superoxide dismutase, U/mL	50.3 ± 2.6^b^	64.6 ± 0.7^a^	75.7 ± 1.3^a^	54.9 ± 2.7^b^
Glutathione, μmol/L	4.0 ± 0.1^b^	4.9 ± 0.2^a^	4.0 ± 0.2^b^	3.8 ± 0.1^b^
Glutathione peroxidase, U/mL	728 ± 24^b^	857 ± 26^a^	878 ± 26^a^	739 ± 25^b^
Malondialdehyde, nmol/mL	6.5 ± 0.1^a^	5.2 ± 0.1^c^	6.0 ± 0.1^b^	5.9 ± 0.1^b^
Intestinal mucosa				
Superoxide dismutase, U/mg protein	7.3 ± 0.2^c^	8.8 ± 0.1^a^	8.7 ± 0.1^a^	7.8 ± 0.1^b^
Glutathione, μmol/g protein	216 ± 12^b^	313 ± 15^a^	312 ± 38^a^	232 ± 15^b^
Glutathione peroxidase, U/mg protein	47.4 ± 3.0^b^	81.5 ± 1.8^a^	76.0 ± 3.8^a^	54.4 ± 3.0^b^
Malondialdehyde, nmol/mg protein	0.84 ± 0.03^a^	0.47 ± 0.06^b^	0.43 ± 0.02^b^	0.79 ± 0.02^a^

1 Values are means ± SEMs, n = 6. Different superscript within a row means significantly different (*P* < 0.05).

2 NC, negative control; GSPs, grape seed proanthocyanidins; AGS, reduced antibiotics + GSPs; PC, positive control.

## DISCUSSION

In the present study, results demonstrated that dietary GSPs can improve diarrhea in early weaning and is comparable to antibiotics. Dietary GSPs also increased the ADG and the F/G ratio, without marked changes in ADFI. Therefore, GSPs treatment was shown to help enhance the feed utilization efficiency and improve the growth performance, which is similar with previous studies [21]. The vast majority of antibiotic substitutes produce inconsistent results relative to comparison to antibiotics [7, 22]. Our findings provide not only a potential effective antibiotic alternative, but also an efficient strategy for partial substitution of antibiotics with other growth promoting substances. Synergistic effects were observed between supplemental GSPs combined with colistin compared with GSPs or colistin used alone [23]. Our data also confirmed that the AGS group (treated with 50% reduced antibiotics + GSPs) has comparable effects to GSPs treatment, which suggest that GSPs can substitute for a half-dose colistin, at minimum. Colistin has become the last line of defense for the treatment of infections, in particular carbapenem-resistant *Enterobacteriaceae* (CRE). However, resistance to colistin, was first identified in China in 2015, and has subsequently been reported from numerous other countries [5-6].

In the present study, results of the lactulose/mannitol test showed that dietary GSPs decreased the intestinal permeability. Previous studies have shown weaning stress results in a sustained impairment of the intestinal barrier, shorter villus, and deeper crypt depth that leads to the deterioration of intestinal morphology [24]. Data of intestinal morphology indicated that villus height in the small intestine of piglets in the GSPs group was much higher than that in the control group, and crypt depth in the GSPs group decreased compared with the control group, and therefore the ratio of villus height to crypt depth (V/C ratio) of each intestinal segments in the GSPs group increased. These data suggested that GSPs treatment may improve the intestinal mucosal barrier function, and help to overcome the intestinal barrier injury and associated piglet diarrhea caused by weaning stress. In order to verify this hypothesis, the expression of several characteristic mucosal barrier proteins, including occludin, claudin-1 and ZO-1 was tested. The results showed that the expression of occludin was increased in GSPs treatment, both in ileum and colon, which was consistent with our hypothesis [25], whereas the expression of claudin-1 and ZO-1 had no difference in different groups, which suggest that occludin may play an role important role in the GSPs treatment.

Mucosal oxidative stress has been identified to be contributed to the progression of weaning diarrhea [26]. It is apparent in this study that the GSPs caused an amelioration in antioxidant status that was reflected in the extra-intestinal environment because of major changes in antioxidant parameters. Glutathione peroxidase is implicated in protecting gastrointestinal mucosal cells against damage from various insults [26]. The malondialdehyde levels in tissues and blood are generally used as biomarkers of endogenous lipid peroxidation and radical-induced damages [27]. Decreased oxidative stress may lead to reduced damages to intestinal mucosal barrier, which subsequently leads to reduced intestinal permeability.

In this research, GSPs increased bacterial abundance and diversity in both ileum and colon, suggesting that GSPs has a significantly positive impact on the biodiversity of the ecosystem of the intestine. An increase in microbial diversity has been related to enhance ecosystem stability and resistance to pathogen invasion [28-29]. Therefore, the increased diversity in the intestinal microbiota by GSPs most likely contributed to the improved intestinal mucosa immune system [30]. Our findings indicate that the most abundant OTUs associated with the colon digesta from the GSPs group were *Firmicutes* (78.25%) and *Bacteroidetes* (20.34%). In phylum level distributions, *Firmicutes* and *Bacteroidetes* were prevalent members of the gut microflora in each treatment. The gut microbiota of pigs mainly consists of the *Bacteroidetes* and *Firmicutes* divisions [31]. *Ruminococcaceae* is normally associated with enhancing feed conversion in piglets due to its cellulose-degrading capacity [32]. *Ruminococcaceae* is also conductive to cumulating more energy from complex polysaccharides which are otherwise resistant to the action of digestive enzymes [33]. These results suggested that GSPs supplementation can increase the number of beneficial bacteria and decrease the number of harmful bacteria at the phylum level.

At the family level, our results demonstrated that GSPs treatment decreased the abundance of *Lactobacillaceae*, whereas the abundance of *Clostridiaceae* was increased in both the ileal and colonic lumen. *Lactobacillus* has been reported to rapidly establish a complex bacterial community, which protect the host from pathogenic bacteria. It is reported that dietary supplementation can significantly increase *Lactobacillus* in animals [34]. However, in the current study, the number of ileal and colonic *Lactobacillus* significantly decreased in the GSPs group. The effects are consistent with previous reports that a decrease in *Lactobacillaceae* in the ileal lumen may reduce loss of energy, since *Lactobacilli* expedites the microbial bile salt hydrolase activity and impairs lipid absorption by the host [35]. Previous studies have shown that *Clostridium* is the dominant bacteria in pig intestines, whose metabolite is butyric acid. Butyric acid is always produced by butyric acid bacteria *via* pyruvic acid pathway. Acetic acid and lactic acid as gut microbial metabolites are also crucial source of butyrate [36].

In addition, GSPs treatment significantly increased the concentration of propionic acid and butyric acid. Notably, three GPRs of VFAs, GPR41, GPR43 and GPR109A have been well documented. From the perspective of VFAs, acetate preferentially activates GPR43, propionate displays similar agonism on GPR41 and GPR 43, and butyrate preferentially activates GPR41 and GPR109A [37]. VFAs combined with their transporters and receptors exert multiple effects on host. Our data showed that the expression of GPR41 in ileum and colon was increased in the GSPs group, which may be due to the increased propionic acid and butyric acid in digesta. On contrast, the expression of GPR43 and GPR109A had no difference in different groups, which suggest that GPR41 may play an role important role in the GSPs treatment. Our findings are consistent with the previous studies, which indicated that GPR41 gene is highly expressed in intestinal tissue and adipose tissue, while GPR43 gene is highly expressed in the hypothalamus and adipose tissue [38]. GPR109A is mainly expressed in adipose tissue, spleen and immune cells [39].

In summary, the current study illustrated that GSPs may benefit weaning piglets as an effective antibiotic alternative. Dietary GSPs reduced the occurrence of diarrhea by decreasing intestinal permeability and improved intestinal morphology. In addition, GSPs treatment increased microbial diversity and improved the microbial community. The increasing concentrations of propionic acid and butyric acid may activate GPR41, which may in turn contribute to the improved intestinal mucosa barrier. Our findings revealed the potential mechanism by which intestinal microbiota mediate the effects of dietary GSPs on mucosal barrier.

## MATERIALS AND METHODS

### Piglets, diets and experimental protocol

All procedures used in this experiment were approved by the Institutional Animal Care and Use Committee of China Agricultural University.

Crossbred weaned piglets (Duroc × Landrace × Large White, *n* = 120, weaned at 28 d) were selected from a pool of 30 litters. After a 3-d adaptation period during which all piglets received the same base diet, the pigs (9.3 ± 0.5 kg of body weight) were assigned to 1 of 4 treatments (*n* = 30 per treatment) stratified by weight and sex. A basal diet was formulated to meet the nutrient requirements for the pigs according to the NRC standards. Dietary treatments included a corn-soybean meal control ration without antibiotics (NC group = negative control), a similar ration with 250 mg/kg GSPs (GSPs group), a similar ration with 50 mg/kg kitasamycin, 20 mg/kg colistin sulfate and 250 mg/kg GSPs (Jianfeng Biology Co, Tianjin, China) (AGS group = reduced antibiotics + GSPs), or with 100 mg/kg kitasamycin and 40 mg/kg colistin sulfate (PC group = positive control).

During the 28 d experiment, pigs were fed their respective diets and allowed ad libitum access to feed and water. The piglets were individually weighed on d 28 and feed consumption per pen was recorded weekly. Fecal consistency was visually assessed at 9:00 h and 16:00 h each day by observers blind to treatments using a modification of the method described by Ma et al. [40]. The occurrence of diarrhea was defined as production of feces at level of 2 or 3 for 2 continuous days.

### Small intestinal permeability

Intestinal permeability was assessed on d 28 prior to weighing using the lactulose to mannitol differential absorption test according to our previous report [20]. Briefly, urine samples of piglets (*n* = 6/treatment, 6 piglets were randomly selected from the total 30 in each group, to conduct this assay and following assays) were collected after 6 h of fasting for baseline urinary sugar measurement. After the samples were obtained, the piglets were intragastrically administered 5 ml of lactulose (0.2 g/mL) and 5 mL of mannitol (0.1 mg/mL). Urinary lactulose and mannitol concentrations were determined by anenzymatic technique [20]. Lactulose/mannitol (L/M) excretion ratios were calculated as an index of intestinal permeability.

### Sample collection

On d 28, 6 blood samples per treatment from the selected piglets were collected from the jugular vein using tubes without anticoagulant (Becton Dickinson, Franklin Lakes, NJ). Blood samples were allowed to clot at room temperature for 20 min and centrifuged at 1,600 × g for 10 min at 4°C. Serum was then removed and stored at -20°C until determination. The selected piglets were sacrificed, the intestinal segments (jejunum, ileum and colon) were obtained, and the mucosa in each segment were separated. The digesta from the ileum and colon were collected. All these samples were stored at -80°C until detection.

### Microbial diversity analysis

Microbial DNA was extracted from each ileal and colonic digesta samples according to manufacturer protocols (Meiji, Shanghai, China). The V4-V5 regions of the bacteria 16S ribosomal RNA gene were amplified by PCR (95 °C for 3 min, followed by 27 cycles at 95 °C for 30 s, 55 °C for 30 s, and 72 °C for 45 s and a final extension at 72 °C for 10 min) using primers 338F 5’-barcode- ACTCCTACGGGAGGCAGCA-3’ and 806R 5'GGACTACHVGGGTWTCTAAT-3’, where the barcode is an eight-base sequence unique to each sample. PCR reactions were performed in triplicate 20 μL mixture containing 4 μL of 5 × FastPfu buffer, 2 μL of 2.5 mM dNTPs, 0.8 μL of each primer (5 μM), 0.4 μL of FastPfu polymerase, and 10 ng of template DNA.

Amplicons were extracted from 2% agarose gels and purified using the AxyPrep DNA Gel Extraction Kit (Axygen Biosciences, Union City, CA, U.S.) according to the manufacturer's instructions and quantified using QuantiFluor™ -ST (Promega, U.S.). Purified amplicons were pooled in equimolar and paired-end sequenced (2 × 250) on an Illumina MiSeq platform according to the standard protocols.

Raw fastq files were demultiplexed, quality-filtered using QIIME (version 1.17) with the following criteria: (i) The 300 bp reads were truncated at any site receiving an average quality score < 20 over a 50 bp sliding window, truncated reads that were shorter than 50bp were discarded. (ii) Inexact barcode matching, 2 nucleotide mismatch in primer matching, or reads containing ambiguous characters were removed. (iii) Only sequences that overlap longer than 10 bp were assembled according to their overlap sequence. Reads which could not be assembled were discarded.

Operational Units (OTUs) were clustered with a 97% similarity cutoff using UPARSE (version 7.1 http://drive5.com/uparse/) and chimeric sequences were identified and removed using UCHIME. The taxonomy of each 16S rRNA gene sequence was analyzed by RDP Classifier (http://rdp.cme.msu.edu/) against the Silva (SSU115) 16S rRNA database using confidence threshold of 70%.

### The concentration of VFAs

The concentrations of volatile fatty acids (VFAs) were determined by a gas chromatographic method according to the procedures of Zhang et al. [41] with modifications. Briefly, about 1.5 g of thawed ileal and colonic digesta was suspended in 1.5 mL of distilled water in a screw-capped tube. The entire sample was centrifuged. Then 1 ml of supernatant was collected to an ampoule and mixed with 200 μL of metaphosphoric acid. The ampoules were placed in an ice bath for 30 min and centrifuged again for 10 min. The sample was injected into a HP 6890 Series Gas Chromatograph (Hewlett Packard, PA, California) equipped with a HP 19091N-213 column with 30.0 m × 0.32 mm i.d. (Agilent, PA, California). The injector and detector temperatures were 185 and 210 °C, respectively.

### Morphological evaluations

Villus height and crypt depth in the jejunum and ileum were determined as previously described [19, 42]. Briefly, these segments were fixed in 4% paraformaldehyde for 24 h, and then embedded in paraffin wax. Sections of 4 μm were cut and stained with hematoxylin and eosin. Measurements for villus height and crypt depth were taken by using the Axioskop-2 Microscope (Olympus, Japan) and the Image Processing System (Visitron Systems).

### Protein extraction and immunoblot analysis

The expression of SCFAs receptors GPR41, GPR43 and GPR109A, as well as mucosal barrier protein occludin, claudin-1 and ZO-1, were determined due to its crucial roles in intestinal mucosal barrier function [19]. The total protein contained in jejunal and colonic tissue samples was extracted. Equal amounts of protein extracts (20 μg) were fractionated on SDS-PAGE and transferred to polyvinylidenedifluoride membranes (Bio-Rad Laboratories). The membranes were blocked with 5% non-fat milk solution and then incubated with diluted primary antibodies. The primary antibodies against GPR41, occludin, claudin-1 and ZO-1 were obtained from Santa Cruz (Santa Cruz, CA), GPR43 from Abcam (Cambridge, MA), GPR109A from Acris (Rockville, MD), as well as β-actin from Sigma-Aldrich (St Louis, MO). After incubation with HRP-conjugated secondary antibody, signals were visualized by the Odyssey Infrared Imaging System (LI-COR Biosciences). Blots analysis was carried out at three replicates per treatment by “Quantity One” Software (BioRad Laboratories), with subsequent calculation of the ratio between the band intensities of each barrier proteins and β-actin.

### Antioxidant parameters

Serum samples were thawed and thoroughly mixed immediately before analysis. Equal amounts of mucosa in different intestinal segments from the same piglet were blended to form a single sample. The blended samples (*n* = 6/treatment) were homogenized in phosphate buffer (10 mM, pH 7.4), and centrifuged at 1,600 × g for 10 min at 4°C. The supernatant was stored at -80°C until further assays. Antioxidant parameters including superoxide dismutase, glutathione peroxidase, glutathione and malonaldehyde in serum and intestinal mucosa were all determined using assay kits according to the manufacturer's instructions (Nanjing Jiancheng Bioengineering Institute).

### Statistical analysis

The difference in the diarrhea incidence among the three treatments was tested by the Chi-Square Contingency Test. All other data were analyzed using SAS Version 9.1. The results were presented as means ± SEMs. One-way analysis of variance followed by Tukey's multiple range tests was used for equal variances. Kruskal-Wallis one-way analysis of variance was conducted to compare means with unequal variances. For all statistical analyses, probability values less than 0.05 were considered significant.

## Abbreviations

AGS: reduced antibiotics + GSPs
GSPs: grape seed proanthocyanidins
SCFAs: short-chain fatty acids
GITs: gastrointestinal tracts
OTU: operational taxonomic unit
NC: negative control
PC: positive control
(ADG) and (ADFI): average daily weight gain and average daily feed intake (ADFI)
VFAs: volatile fatty acids
V/C: villus height to crypt depth
GPR: G-protein coupled receptor
ZO-1: zonula occludens protein-1.
